# Mental health practitioners’ experiences and practices in making decisions about onward care for patients presenting to emergency departments with self-harm or suicidal ideation: systematic review and meta-synthesis

**DOI:** 10.1192/bjo.2026.11007

**Published:** 2026-03-30

**Authors:** Mimi Suzuki, Alexandra E. Bakou, James Dove, Rose McCabe

**Affiliations:** School of Health and Medical Sciences, City St George’s, University of London, UK; Hospital Division, North London NHS Foundation Trust, London, UK

**Keywords:** Self-harm, emergency department, suicide, psychosocial assessment, liaison psychiatry

## Abstract

**Background:**

Emergency department mental health practitioners (MHPs) decide onward care for individuals presenting with self-harm or suicidal ideation. However, their experiences and practices in making these decisions remain underexplored.

**Aims:**

To synthesise research on MHPs’ experiences and practices in making decisions about onward care for patients presenting to emergency departments with self-harm or suicidal ideation.

**Method:**

We searched six databases (inception to July 2024) for empirical studies of MHPs making care decisions for self-harm or suicidal patients in emergency departments. We used a segregated mixed-methods design, applying narrative synthesis of quantitative data and thematic synthesis of qualitative data.

**Results:**

Eleven studies were included (one quantitative, one mixed-methods, nine qualitative). Narrative synthesis of quantitative data produced two themes: (a) subjective decision-making and variability among MHPs and (b) impact of the institutional mandate to discharge within 4 h on referral outcomes. Thematic synthesis of qualitative data generated five themes: (a) risk-centric culture is anti-therapeutic and shapes defensive practice, scepticism toward patients and burnout; (b) time and environmental pressures impact therapeutic potential of assessments; (c) ‘battling’ to access services: gatekeeping, cycles of repeat attendances affecting patient safety and staff moral injury; (d) strategies to facilitate access and extending care to overcome challenges in the emergency department and (e) potential for training to counter negative attitudes and stereotypes.

**Conclusions:**

Intersecting institutional, systemic and emotional pressures shape MHPs’ practices, undermining assessment quality and access to care. System-level reforms and training should promote relational, compassionate care. Limited quantitative evidence restricted integration, and the review reflects high-income Western settings.

Emergency departments serve as critical touchpoints for individuals experiencing mental health crises. Presentations involving self-harm (an intentional act of self-poisoning or self-injury, irrespective of the motivation or apparent purpose of the act) and suicidal ideation are associated with an increased risk of subsequent suicide.^1,2^ These presentations occur in settings characterised by high patient volumes, limited time and resources, and complex clinical demands.^3^

Mental health practitioners (MHPs) working in emergency departments, typically as part of specialist liaison mental health teams embedded within the department, conduct psychosocial assessments to evaluate patients’ needs and risks and make decisions about onward care. Although conceptually distinct, suicidal ideation and self-harm are routinely managed through similar processes in the emergency department.^4^ Evidence indicates that comprehensive psychosocial assessments can reduce the likelihood of future self-harm,^5^ facilitate timely follow-up^6^ and improve engagement with mental health services.^7^

## Existing literature

Existing reviews highlight several challenges in the assessment and management of people experiencing self-harm or suicidal ideation. One review shows variability in suicide risk assessment practices, with MHPs relying on individual judgement despite the availability of structured tools. These approaches often marginalise patient perspectives, and risk assessments adopt narrow formulations that fail to capture the complexity of suicidal distress.^8^ In emergency departments, a recent review found that decision-making is influenced by staff attitudes, confidence and knowledge; patient characteristics such as method of self-harm, age and gender; and contextual factors including service and staffing availability.^9^ Earlier work has also documented negative or ambivalent attitudes among clinical staff toward people who self-harm, and highlighted the consequences for engagement and quality of care.^10^

These reviews revealed three key gaps. First, although existing reviews examine emergency department decision-making, none focused exclusively on MHPs, who are primarily responsible for conducting psychosocial assessments and determining onward care. Second, existing evidence identifies factors associated with decision-making, but offers limited insight into how these operate in practice. The interactional practices and communication through which risk is assessed and decisions are made remain unexamined. Third, although emergency department-specific contextual factors have been identified, they do not examine their impact on MHP experiences and practices.

Addressing these gaps is crucial to understanding how decisions about care are made, and how contextual factors shape outcomes for patients presenting with self-harm or suicidal ideation. The aim of this systematic review is to focus on MHPs to explore: What are MHPs’ experiences and/or practices when making decisions about onward care for patients presenting to emergency departments with self-harm or suicidal ideation?

## Method

A mixed-methods review was conducted following Sandelowski et al.^11^ The protocol was registered with PROSPERO (identifier: CRD42024504583, date of registration: 10 October 2024, date of extraction: 27 November 2024). This systematic review is reported according to the Preferred Reporting Items for Systematic Reviews and Meta-Analyses (PRISMA) checklist.

Ethical approval was not required as this systematic review synthesises data from previously published studies and contains no identifiable personal information.

### Eligibility criteria

Studies were included if they met the following criteria.

- Studies that were primary empirical studies.
- Study participants were MHPs involved in emergency department psychosocial assessment and onward care decisions, including psychiatrists and non-medical clinicians (e.g. mental health nurses, social workers). We did not exclude studies on the basis of professional group because liaison emergency department mental health services are typically multidisciplinary, and included studies rarely reported findings disaggregated by role. Findings are presented as relating to emergency department MHPs collectively. Studies with physical health professionals as participants in addition to MHPs were included if results could be separated so that only data from MHPs were included.
- We focused MHPs serving adult emergency department populations. Studies exclusively focused on paediatric or adolescent populations were excluded. Studies based in general hospital emergency departments were included; studies set exclusively in psychiatric emergency services were included only if they were emergency department-linked services.
- Studies on MHPs’ views, experiences or practices of decision-making processes or mental healthcare processes in emergency departments, such as decisions about onward care (e.g. hospital admission, discharge to primary care, referral to community mental health services), risk assessments in emergency department settings and care pathways for adults presenting to emergency departments for self-harm or suicidal ideation.
- Studies that were reported in English or with a full-text English translation.

Studies were excluded if they focused on statistical predictors not exploring decision-making processes.

### Information sources

Six electronic databases were searched from database inception until July 2024, because of their nursing, medicine and mental health research coverage: MEDLINE, Embase, EMCare, PsycINFO, CINAHL and Web of Science.

### Search strategy

A search strategy was developed focusing on five key areas: (a) suicidal ideation and self-harm; (b) MHPs; (c) emergency department setting; (d) experiences and practices of MHPs; and (e) decision, assessment or management. The search strategy was developed, piloted and refined in MEDLINE before being adapted for each database (see Supplementary File 1 available at https://doi.org/10.1192/bjo.2026.11007). MeSH terms were run in combination with free-text searches of titles and abstracts.

Search records were exported to Zotero (version 7.0.32 for macOS; Corporation for Digital Scholarship, Vienna, Virginia, USA; https://www.zotero.org/) and manually de-duplicated. M.S. screened titles to exclude irrelevant retrievals (e.g. assisted-dying studies). Remaining studies were exported to Rayyan (web application originally released 2014 used on macOS; Qatar Computing Research Institute (QCRI), Hamad Bin Khalifa University, Doha, Qatar; https://rayyan.ai), an online systematic review platform. M.S. and A.E.B. independently screened titles and abstracts. In cases of disagreement, the study advanced to full-text screening. M.S. and A.E.B. independently screened full texts against inclusion criteria, with disagreements resolved through discussion and consensus. Interrater reliability for screening was calculated using percentage agreement and Cohen’s kappa (*κ*). Reasons for exclusion were article type, participant, setting, condition (i.e. not suicidal/self-harm patients in the emergency department) or domain (i.e. not MHPs’ views, experiences or practices of decision-making processes).

### Data extraction

M.S. and A.E.B. independently extracted the following variables: country, study design and aims, sample size, description of practitioner roles, age and gender, description of patients (i.e. related to self-harm only, suicidal ideation only, or self-harm and/or suicidal ideation) and main findings. For categorical variables, interrater reliability was calculated using Cohen’s kappa (*κ*). For continuous variables, intraclass correlation coefficient (ICC) was computed. Free-text fields including study aims and main findings were also independently extracted, and discrepancies were resolved through discussion. For qualitative studies, following Thomas and Harden’s approach,^12^ data were extracted for all text labelled as results/findings.

### Quality assessment and assessment of certainty/confidence in review findings

Study quality for individual studies was independently assessed by M.S. and A.E.B., using the Mixed Methods Appraisal Tool (MMAT^13^). The MMAT includes two screening questions and additional questions dependent on study design. Interrater reliability was calculated using Cohen’s kappa (*κ*) across all MMAT criteria. Disagreements were resolved through discussion and consensus. Low-quality studies remained in the review and MMAT ratings facilitated interpretation.

We used the Grading of Recommendations Assessment, Development and Evaluation (GRADE)^14^ and Confidence in the Evidence from Reviews of Qualitative Research (GRADE-CERQual)^15^ approach to summarise review findings into discrete statements. Confidence in review findings were independently assessed by A.E.B. and M.S., using the GRADE approach for quantitative findings and GRADE-CERQual for qualitative findings.^14–16^ Final assessments were based on consensus between A.E.B. and M.S.

### Data synthesis

**Fig. 1:**
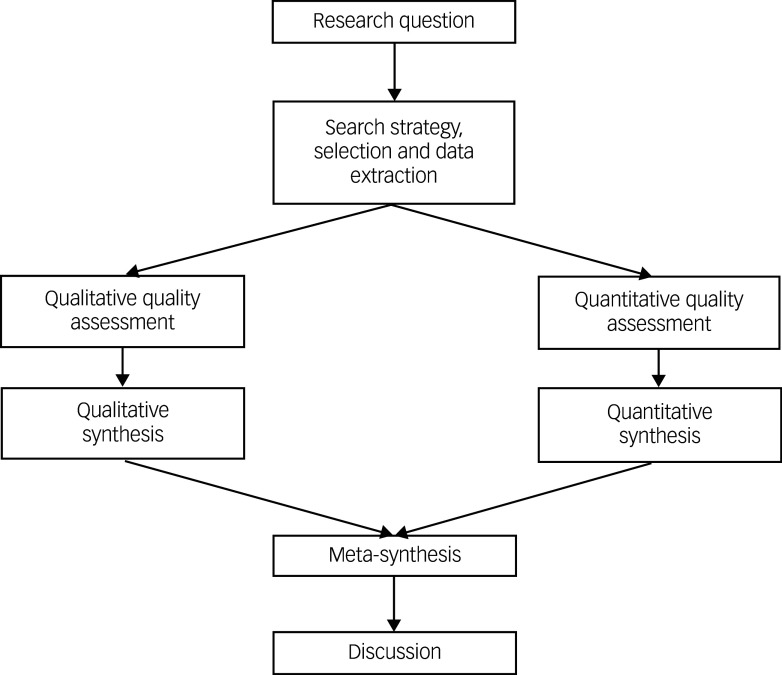
Flowchart of segregated mixed-methods design.

A segregated approach^11^ was adopted where qualitative and quantitative findings are viewed as complementary (Fig. 1). Thus, qualitative and quantitative findings were synthesised separately and meta-synthesis of both syntheses were conducted at an interpretive level, highlighting convergence and divergence between qualitative and quantitative studies.

Quantitative findings were synthesised using narrative synthesis,^17^ and qualitative findings were synthesised using thematic synthesis.^12^ Meta-analysis were considered for quantitative evidence. For thematic synthesis, line-by-line coding^18^ of qualitative findings was conducted on NVivo (version 14 (2023) for macOS; QSR International Pty Ltd, Melbourne, Victoria, Australia; https://www.qsrinternational.com/nvivo-qualitative-data-analysis-software). Codes were analysed using the constant comparative method to develop descriptive themes, followed by analytic themes and, finally, broader overarching themes.

A visual model was developed from the meta-synthesis through cross-comparison across themes and team discussions with the authors (doctoral researcher, postdoctoral researcher, professor with expertise in qualitative research and a consultant emergency department liaison psychiatrist).

## Results

**Fig. 2:**
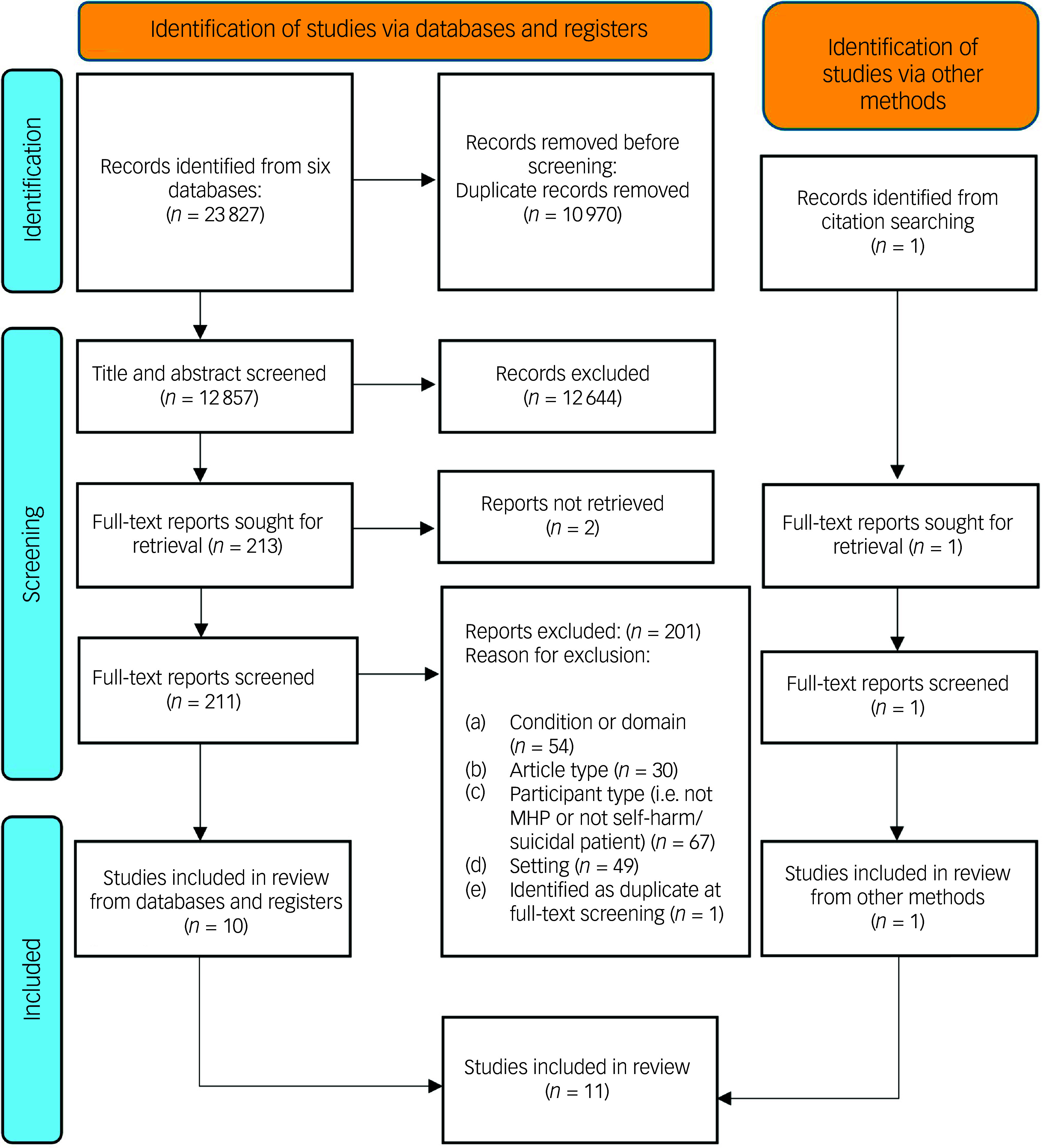
Preferred Reporting Items for Systematic Reviews and Meta-Analyses flow diagram. MHP, mental health practitioner.

Eleven studies were included (see Fig. 2 for PRISMA flow diagram): one mixed-methods study,^19^ one quantitative study^20^ and nine qualitative studies, six of which were interview/focus group studies^21–26^ and three of which were conversation analytic studies analysing professional–patient communication.^27–29^ Supplementary Table 1 presents a summary of included studies dating from 2015. There were seven studies from the UK, one from Ireland, one from USA and two from Australia. Five studies focused on patients presenting with self-harm only, five studies included patients presenting with suicidal ideation and/or self-harm and one study focused on patients with multiple self-harm emergency department presentations (described as ‘repeat self-harm’).

Interrater reliability was high. Agreement during title and abstract screening was 99% (*κ* = 0.90, 95% CI 0.85–0.95). Full-text screening yielded 68% agreement (*κ* = 0.28, 95% CI 0.06–0.51).

For data extraction, categorical variables (country, study design, description of practitioner roles, age and gender, description of patients) demonstrated substantial (*κ* = 0.70, 95% CI 0.41–0.99) to perfect agreement (*κ* = 1.00). Continuous variables showed excellent consistency: interrater reliability for the sample size was high (ICC = 0.98), and percentage female exhibited perfect numerical and categorical agreement (ICC = 1.00; *κ* = 1.00).

### Quality assessment and confidence in review findings

All qualitative studies met all five quality criteria, demonstrating alignment between research questions, methodology, data collection and interpretation. However, the qualitative component of Phillips et al’s^19^ mixed-methods study was of poorer quality. The one quantitative study was of low quality, as was the quantitative component of the mixed-methods study. A full breakdown of MMAT ratings for each study is in Supplementary File 2. Agreement for study quality assessment across MMAT criteria was high, with 95% agreement (*κ* = 0.92, 95% CI 0.84–1.00).

Certainty all three quantitative review findings were judged to be of very low certainty, using the GRADE approach. A summary of quantitative findings is presented in Supplementary Table 2. Our assessment for each component of GRADE is shown in the evidence profiles (Supplementary File 3). We used the GRADE-CERQual approach to grade our confidence in 16 qualitative review findings. A summary of the qualitative findings is presented in Supplementary Table 4. We graded 11 studies as high confidence and five as moderate confidence. Our main concerns were related to the relevance of the finding, as all studies were conducted in a high-income, Western context. Some findings had contributing studies from only the UK context. Our explanation of the GRADE-CERQual assessment for each review finding is shown in the evidence profiles (Supplementary File 4).

### Quantitative synthesis

Quantitative components from two studies were included. Meta-analysis was not conducted because of limited quantitative evidence. Differences in focus meant that a combined synthesis was not feasible, and findings are reported separately. The quantitative findings highlighted two issues influencing MHPs decisions about onward care.

#### Subjective decision-making and variability among MHPs

Phillips et al^19^ conducted a vignette-based survey to explore discharge decisions following self-harm. Participants assessed suicidal intent and discharge decision across nine hypothetical cases. Responses were broadly categorised as admission or no admission. The study found substantial variability in decisions even when participants agreed on suicidal intent. For instance, in one vignette depicting an impulsive but socially supported female whom 94% of participants judged to have suicidal intent, only 58% recommended admission. Conversely, in a vignette involving a socially isolated man with substance use issues, 96% recommended admission despite most not viewing the act as suicidal. Social support appears to influence discharge decisions beyond clinical risk.

#### Impact of institutional mandate to discharge within 4 h on referral outcomes

Haslam and Jones^20^ analysed routine data from a liaison mental health to assess whether a breach of the 4 h emergency department discharge target before referral to the team affected whether patients were discharged from the hospital or were not discharged and received one of the following: admission to a psychiatric unit, referral for further Mental Health Act assessment or referral to a crisis decision unit (CDU) – a short-stay unit where a patient receives further observation, assessment or support. There was no significant association between breach of the 4 h emergency department target and whether patients were discharged (*χ* ^2^(1) = 0.091; *p* < 0.763). However, patients seen within 4 h target were more often referred for Mental Health Act assessment, whereas those seen later were more often referred to a CDU, which is a comparatively less invasive and restrictive option for patients. The authors suggested time pressure may shape referral pathways, although statistics were not provided.

### Qualitative synthesis

Five overarching themes were identified across ten analytic themes derived from 35 descriptive categories, detailed in Table 1.

**Table 1:** Qualitative synthesis: summary of overarching themes and analytic themes

Overarching themes	Analytic themes	Descriptive categories
Theme 1: Risk-centric culture is anti-therapeutic and shapes defensive practice, scepticism toward patients and burnout	‘It’s risk, risk, risk’: dominance of risk and erosion of therapeutic engagement	Focus on risk overshadows therapeutic engagement Narrow definitions of risk that do not capture patient’s perspective of risk Scepticism arising from discrepancies in patients accounts, suspecting malingering and implying implausibility Risk is ambiguous and some MHPs are not confident in decisions More experienced MHPs trust ‘gut feeling’
	Defensive practices to protect from liability	Fear of liability especially during night shifts Defensive practices such as narrow definition of risk, documentation aimed at demonstrating accountability and shifting responsibility of care Defensive posture can lead to negative attitudes, especially toward patients presenting with ‘inappropriate’ presentations
Theme 2: Time and environmental pressures affect therapeutic potential of assessments	Inadequate emergency department environment for therapeutic psychosocial assessments	Quality of assessment affected by chaotic, busy emergency department MHPs attempt to find solutions to improve therapeutic environment, but that adds to time pressure
	Pressure to discharge compromising comprehensive assessments or else longer wait times for other patients	No time for thorough assessments Four-hour target not appropriate for complex presentations Concern about knock-on effect of increasing waiting times in chaotic emergency department for other patients Documentation adds to time pressure but detailed narrative documentation important
Theme 3: ‘Battling’ to access services: gatekeeping, cycles of repeat attendances affecting patient safety and staff moral injury	Barriers to access and patients falling through the cracks in the system	Restrictive eligibility criteria when referring patients to both secondary and primary care services (e.g. secondary care have a high risk threshold; exclude because of lack of diagnosis, current alcohol use, being in touch with more than one service; exclude if crises are attributable to ‘social’ or ‘relationship’ factors; exclude if crises are considered ‘acute’) Fragmented system of two-way gatekeeping from other services with redundant repeat assessments and access to services varied by who picks up the phone The responsibility is placed back on the patient (offering self-control/self-help as reasonable alternative to mental health services) Exclusionary referral criteria affected staff morale and being placed as gatekeepers or bearers of bad news led to moral injury
	Cycles of repeat attendances: constrained care and moral injury in the absence of support to address root causes in wider mental health system	MHPs view lack of timely community-based intervention underlie to cycles of repeat attendances MHP can only provide a band aid, but need to address root causes of self-harm and suicidal ideation Frustration with fragmented and disjointed wider mental health system Inability to offer more led to feelings of futility, and desensitisation as a protective response Inadequate formal support for MHPs despite the need, and turning to informal support within team
Theme 4: Strategies to facilitate access and extending care to overcome challenges in the emergency department	Facilitating access to mental health services: open communication with services and tailored referrals letters to advocate for patient access	Knowledge of referral criteria and input from multidisciplinary teams facilitated referrals Senior support from consultants and clinical psychologists facilitated access to specialised mental health services Building strong relationships with other services helped streamline referrals Tailoring referral letters to align with specific criteria
	Out-patient clinics: extending the care pathway for comprehensive assessment outside of emergency department constraints	Out-patient clinics to provide continuity of assessment and care Out-patient clinics were not available to all liaison mental health services Out-patient clinics have their own entry criteria
Theme 5: Potential for training to counter negative attitudes and stereotypes that are still prevalent	MHPs recognised their own attitudes as empathetic, compassionate and understanding, yet still hold negative attitudes and stereotypes	MHPs recognised self-harm as coping mechanism Negative language and stereotypes were still prevalent in MHPs’ responses and practices MHPs suggested these negative attitudes were passed down from senior members of the team
	Training as a mechanism for attitudinal change and increased confidence	MHPs are keen to continue to improve their ability to care for patients who present with self-harm or suicidal ideation Increased training was linked to improved confidence and reduce negative stereotypes Current mental health training programmes left new MHPs feeling unprepared

MHP, mental health practitioner.

### Theme 1: risk-centric culture is anti-therapeutic and shapes defensive practice, scepticism toward patients and burnout

#### ‘It’s risk, risk, risk’: dominance of risk and erosion of therapeutic engagement

Four studies^21,22,24,26^ explored risk assessment in practice. Although MHPs incorporate observations, protective factors and collateral information in their assessments,^21^ they described a primary focus on identifying and managing suicide risk:

‘…with the work we do, our head is always thinking its risk, risk, risk. When you think of risk how do you mitigate those risks, that’s the way we think.’ (^24^, p. 4)

Many MHPs expressed discomfort with how risk assessment relied on narrow definitions of risk that failed to capture patients’ own views of what they felt was most risky.^24^ MHPs across two studies^24,26^ noted how the focus on risk was a missed opportunity for therapeutic engagement with patients:

‘…we perhaps undervalue […] how therapeutically beneficial [psychosocial assessments] can be. We do a risk assessment, but actually the interview itself is quite therapeutic and beneficial, and we tend to forget [that] because we don’t see them again.’ (^24^, p. 7)

Risk formulations were shaped not only by patients’ accounts but also alignment with MHPs’ impressions. When there was a discrepancy, MHPs became sceptical, ‘suspected malingering’^21^ and adopted an interrogative stance, probing for inconsistencies or future orientation. Bergen et al^27^ found that some MHPs questioned patients’ credibility, implying implausibility by juxtaposing contradictory statements (e.g. ‘You said you were coping okay before…’) or framing suicidal behaviour as impulsive rather than sustained suicidal intent (e.g. ‘So it was more of an impulsive thing, at the time?’).

Many MHPs spoke of challenges navigating the complexity of suicide risk. MHPs described feeling ‘anxious’, ‘weary’ and ‘fearful’ about making decisions about discharge based on uncertain suicide risk (^22^, p. 317). Others coped with the psychological burden through adherence to assessment protocols, which provided emotional assurance:

‘If I’ve done my due diligence, I feel fine with it, even if there’s a bad outcome. And the risk assessment helps to sort of back you up by saying yes, I asked all these questions.’ (^21^, p. 8)

Some MHPs relied on ‘gut feeling’ rather than always being able to empirically determine the level of risk or appropriate actions to take’ (^26^, p. 5). Clinical experience of working with suicidal patients was seen as key, and accuracy of risk assessments were thought to improve with experience.^21^

#### Defensive practices to protect from liability

Three studies^21,22,24^ described how fear of being held liable for a patient suicide, particularly in the context of a coroner’s inquest, underlie defensive practices that avoid risk of blame. Concerns about liability that underlie these practices were intensified during night shifts, when MHPs lacked support from consultants and there was a higher volume of patients ‘often alone with risks they were not comfortable or confident taking’ (^22^, p. 319). They were further amplified by a broader expectation that MHPs should be able to prevent all suicides:

‘You can’t prevent every suicide. You try and do your best… psychiatry is the only specialty that isn’t allowed to have a death rate.’ (^21^, p. 8)

In this context, fear of liability underpinned a range of defensive practices. These practices included narrowing the definition of risk, relying on extensive documentation, using constrained questioning to generate definitive answers, and shifting responsibility to other services.

MHPs often narrowed the framing of risk by focusing on acute rather than chronic concerns. Patients perceived to have chronic risks was viewed as beyond the scope of the emergency department because it required follow-up that the MHPs could not provide.^21^ At the same time, MHPs expressed uncertainty around what constituted acute risk and were concerned that its definition varied depending on the clinical and legal context:

‘I really think we ought to define acute risk… Everyone I see is at acute risk. They’ve had something go wrong [but] I don’t think they’re going home to kill themselves in a week. That’s why I’m discharging them home. Acute is a word that’s stretched a lot of ways depending on whether [you’re] a malpractice attorney or psychiatrist.’ (^21^, p. 7)

Documentation was important to protect themselves from potential blame, especially in the event of patient suicide. MHPs reported relying on detailed, time-consuming documentation to demonstrate they fulfilled their professional responsibilities.^24^ McCabe et al.^29^ examined communication in psychosocial assessments. The study showed that these records were built on constrained questioning in assessments. MHPs used closed yes/no questions designed to elicit definitive responses about patients’ future thoughts, actions or risk of suicide, even though it did not reflect the complexity of suicide risk. These strategies served not only to assess risk, but to produce a concrete record that could justify decisions if later scrutinised.

Responsibility was often shifted across service boundaries. One MHP reported anxiety about the post-discharge care gap, the period between their assessment and the patient’s next psychiatric appointment, during which they were still legally liable.^21^ Within this context, MHPs described how physical health providers in the emergency department or other mental health services in the community had a ‘fear of taking ownership’ of patients, resulting in responsibility being shifted to the MHPs in the emergency department (^22^, p. 317):

‘You can speak to [General Hospital Trusts providing predominantly physical healthcare] and say, “well, you’re a massive provider of mental health services” and they’d be like “oh no”, almost in denial they play a role and just think that it’s the Mental Health Trust’s issue.’ (^22^, p. 317)

Some MHPs acknowledge that this defensive posture could lead to negative attitudes toward patients, particularly when presentations were seen as inappropriate for the emergency department. McCarthy et al reported that MHPs sometimes blamed or invalidated patients’ help-seeking and ‘[found] reasons why they shouldn’t be [in the emergency department]’ (^22^, p. 317).

### Theme 2: time and environmental pressures affect the quality and therapeutic potential of assessments

#### Inadequate environment for therapeutic psychosocial assessments

The physical environment of the emergency department was highlighted by MHPs as unsuitable for managing patients presenting with self-harm or suicidal ideation in three studies.^22,24,26^

In this busy, chaotic setting, privacy was scarce, making it difficult for MHPs to engage in open, thorough conversations:

‘I don’t think people should be getting reviewed and asked to talk about their deepest, darkest fears and insecurities or whatever, when everyone around them can hear it.’ (^26^, p. 4)

MHPs in McCarthy et al’s study^22^ noted that the emergency department environment impacts their ability to deliver effective and timely care, as they must find a quiet and private room before they can begin the assessment. MHPs felt forced to find solutions within these constraints, such as creatively using limited private spaces to make the best of an inadequate environment.^26^ MHPs felt the emergency department was not a suitable space for people who were presenting with suicidality, and felt it should not be the only place to manage crises, especially if they do not have physical health issues that need to be addressed.^22^

#### Pressure to discharge compromising comprehensive assessments or resulting in longer wait times for other patients

MHPs in three studies^21,22,26^ reported limited time to conduct comprehensive psychosocial assessments. In the UK, the mandated 4 h discharge target compounded this pressure. Likewise, in the USA context, Chunduri et al^21^ described the tension between the institutional pressure to discharge patients quickly to maintain patient flow and the need to conduct a comprehensive assessment. Time constraints compromised the quality of care, leading to rushed assessments, limited care planning and potentially increased repeat emergency department presentations.^22,26^ MHPs found the time pressure particularly challenging for complex presentations, forcing them to rush decisions about discharge or onward care.^22,26^

A key tension identified was the trade-off between spending adequate time with each patient for a comprehensive assessment and managing long waits for others.^22,26^

‘… The legislation mandates that they are seen within 4 h… generally, it’s a cursory assessment … rather than any kind of compassionate care.’ (^26^, p. 4)

Extensive documentation also added to time pressures. Some MHPs voiced ‘irritation’ at spending twice as long writing notes as with the patient themselves,^21,24^ describing it as ‘cumbersome without providing worthwhile clinical benefit’ (^21^, p. 8). However, other MHPs emphasised the importance of detailed narrative documentation to communicate clinical reasoning:

‘When I look at notes, I don’t look at one-line answers, I look for a paragraph as the most helpful. Are you documenting […] decisions in a way that your colleagues that are going to use this chart in the future can take advantage of it?’ (^21^, p. 10)

### Theme 3: ‘battling’ to access services: gatekeeping, cycles of repeat attendances and moral injury

#### Barriers to accessing services and patients falling through the cracks in the system

MHPs described encountering barriers to accessing services for patients presenting with self-harm or suicidal ideation.^24,25^ A recurring issue was the restrictive entry criteria to primary and secondary care. Secondary care access was limited to those with severe and enduring mental illness or high levels of need. Patients without a formal psychiatric diagnosis were often excluded.^25^ Perceptions of patient choice and capacity reinforced entry thresholds:

‘A mental health team would say that person is making their own decision, do they have an underlying mental health issue? – No, that’s a choice they are making.’ (^25^, p. 2)

Other common exclusion criteria included alcohol use, involvement with multiple services and the nature of the presenting crisis. MHPs reported that crises were informally categorised as ‘social’, ‘relationship’ or ‘psychiatric’, with only the latter considered appropriate for secondary care.^25^ Acute or situational crises were deprioritised under the assumption they would resolve without further input.^25,28^ MHPs noted that some patients felt compelled to escalate self-harm to access services:

‘We’re almost reinforcing a situation where people need to continue to self-harm before we will hear what they are saying.’ (^25^, p. 4)

MHPs identified significant gaps in care for patients deemed too high risk for primary care, but not high enough risk for secondary care. In some cases, patients were reclassified as high risk and removed from waitlists if they self-harmed.^25^

Two studies^21,25^ reported MHPs’ frustrations with redundancy of repeated assessments and referral rejections that led to delays in care and undermined the emergency department psychosocial assessment. Acceptance of referrals varied unpredictably depending on who answered the telephone:

‘It depends who answers the phone to who gets accepted basically […] Because different teams have different personalities and different thresholds to what they’ll accept.’ (^25^, p. 4)

Gatekeeping was also observed in MHPs’ communication practices during psychosocial assessments.^28^ MHPs redirected patients to focus on self-control and coping strategies and suggested self-help, informal social support or continue existing plans even when patients asked for more help.

Similarly, Quinlivan et al’s study^25^ reported MHPs expressed moral distress and frustration at being placed in the position of denying care or ‘guarding’ access to scarce services such as in-patient beds:

‘Staff indicated that high caseloads and reduced bed capacity resulted in a focus on risk, gatekeeping (i.e. restricting access to services) and preventing inpatient admission.’ (^25^, p. 3)

This led to moral injury, where MHPs were forced to deliver bad news and navigate ethically distressing decisions under resource constraints, affecting both patient safety and staff morale.^25^

#### Cycles of repeat attendances: constrained care and moral injury in the absence of support to address root causes in wider mental health system

MHPs described a cycle of repeat emergency department attendances caused by the absence of timely community-based treatment.^24,25^ Although MHPs recognised that trauma and psychosocial drivers underpinned self-harm and suicidal ideation, addressing these root causes was seen as beyond their remit and unfeasible given the lack of ‘adequate manpower’ (^22^, p. 317):

‘I feel like, in the emergency department at least, we sort of put band aids on […] problems that can’t be solved in the emergency department.’ (^26^, p. 6)

MHPs emphasised that meaningful, long-term outcomes required broader social reform:

‘“How can we help you?” My gut feeling is how do we reform the social situation that you were brought up in to lead you to this? We can’t, so therefore we will see you again next week… For some of them it’s a very revolving door feeling.’ (^26^, p. 6)

MHPs across four studies^22,24–26^ felt unable to ‘offer more to people’^24^ in the emergency department and ‘felt defeated’^25^ by the lack of adequate aftercare.

Repeat attendances of the same patients compounded MHPs’ feelings of futility and powerlessness and contributed to growing cynicism. As one MHP reflected:

‘… one of the first signs of burnout is not really caring about your patients anymore, so that’s the danger here […] you just get fed up with people rocking up time and time again self-harming.’ (^24^, p. 7).

MHPs described desensitisation and detachment as protective responses:

‘I don’t think people are bad people, I think people just get burnt out and it’s just frustration… It is a big problem, and the thing is just that once people start to burn out, their own self-defences have to kick [in], so people get cynical, and they get desensitised.’ (^26^, p. 6)

Despite these challenges, MHPs received limited pastoral or mental health support. One MHP explained how this is linked to poor staff retention and burnout:

‘… in terms of our own mental health, like what I have noticed is there’s not a lot of staff retention, there’s a lot of burnout, and there’s a lot of people moving from job to job. And I think it’s a no brainer that that’s very much a symptom of a service that isn’t supporting the people.’ (^26^, p. 6)

Instead, MHPs relied on each other to cope with the emotional toll. Informal peer support and teamwork were vital to manage the emotional demands of their work.^22,26^

### Theme 4: strategies to facilitate access and extending care to overcome challenges in the emergency department

#### Facilitating access to mental health services: open communication with services and tailored referral letters to advocate for patient access

MHPs identified clear and well-defined referral criteria in secondary services and input from multidisciplinary staff facilitated access and strengthened emergency department assessments and decisions about onward care.^21,25^ Where direct input from multidisciplinary professionals was not possible, documentation was an important source of information and MHPs wanted more meaningful notes to inform decisions.^21^ Involving consultants or clinical psychologists helped expedite referrals that might otherwise face resistance especially to specialist services.^25^ However, these were contingent on local commissioning.

MHPs also worked to build strong relationships with community services and maintain open communication to streamline referrals and negotiate flexibility regarding eligibility criteria.^25^ MHPs strategically advocated for patients by tailoring referral letters using targeted language that aligned with service thresholds:

‘I’m going to try and fluff the lines as much as I can, to try and help them.’ (^25^, p. 4)

However, this could create situations in which MHPs retract from positions they originally advocated. One MHP described initially exaggerating risk to pursue admission, then downplaying it to justify discharge when the referral was rejected.^21^

#### Out-patient clinics: extending the care pathway for comprehensive assessment outside of emergency department constraints

One study identified a novel out-patient clinic model to overcome challenges of conducting psychosocial assessments in emergency departments.^25^ These clinics extended the care pathway and allowed more comprehensive assessments enabling MHPs to strengthen care plans and support recovery beyond acute episodes. Some MHPs also accepted patients on behalf of crisis teams or facilitated re-contact through crisis lines providing continuity of care and a safety net post-emergency department, so that patients were not discharged into a void without support. MHPs reported feeling more connected to patients’ longer-term recovery, witnessing improvement over time and helping to prevent reattendance.

‘[MHPs] felt that offering interventions from [liaison mental health team] broke the persistent cycle of overassessment and frequent signposting to ill-equipped services.’ (^25^, p. 5)

However, such models are rare, and these clinics often had their own narrowly defined entry criteria. MHPs acknowledged that even with these facilitative strategies, patients continued to fall through gaps in the system.

### Theme 5: potential for training to counter negative attitudes and stereotypes that are still prevalent

#### MHPs recognised their own attitudes as empathetic, compassionate and understanding, yet still hold negative attitudes and stereotypes

MHPs described their attitudes as empathetic and trauma-informed, recognising self-harm as a coping mechanism.^22,23^ Abruptly discouraging self-harm was seen as potentially harmful when it was the patient’s only way of managing distress:

‘I wouldn’t immediately discourage someone to stop self-harming if it is their sole coping mechanism as that can be dangerous, unless of course it’s lethal and could possibly end their life.’ (^23^, p. 1149)

MHPs explained the trauma behind self-harm helping patients feel ‘a sense of relief’ and ‘better understood’ (^23^, p. 1148).

However, negative language and stereotypes persisted. Some MHPs referred to patients’ self-harm as a way to get attention or influence care, ‘she did it because I couldn’t give her the attention she needed right away’ (^23^, p. 7), or framed behaviours as a ‘cry for help’.^22^

‘I see some people who are very unwell, and it is not their fault whereas those who self-harm without being unwell, it was their choice to do that or they did it for a specific gain.’ (^23^, p. 6)

One paper found that these stereotypes reflected attitudes from senior staff and embedded in broader workplace culture:

‘you’ve still got old management style, structures, cultures, behaviours and values that mental health patients have no right coming into A&E [accident and emergency].’ (^22^, p. 317)

#### Training as a mechanism for attitudinal change and increased confidence

MHPs expressed a strong need for training to improve care: ‘I want to know more, I continually want to update my skills and knowledge, how best I can help them’ (^23^, p. 1150). MHPs saw not knowing patient outcomes as a missed learning opportunity:

‘It helps you navigate future care […] how do you learn if not by your mistakes?’ (^21^, p. 9)

MHPs linked training to increased confidence and the ability to challenge stigma bother personally and among colleagues:

‘With that extra bit of education, you would feel more comfortable in your role and you would have more confidence working with [people who self-harm] but also educating colleagues about it’ (^23^, p. 1150)

Greater experience could shift perceptions of self-harm from attention-seeking to a sign of distress.^23^ However, mental health training in nursing programmes was described as too general, leaving MHPs unprepared. One student nurse said the there was little relevance to emergency settings.^22^

### Meta-synthesis

**Fig. 3:**
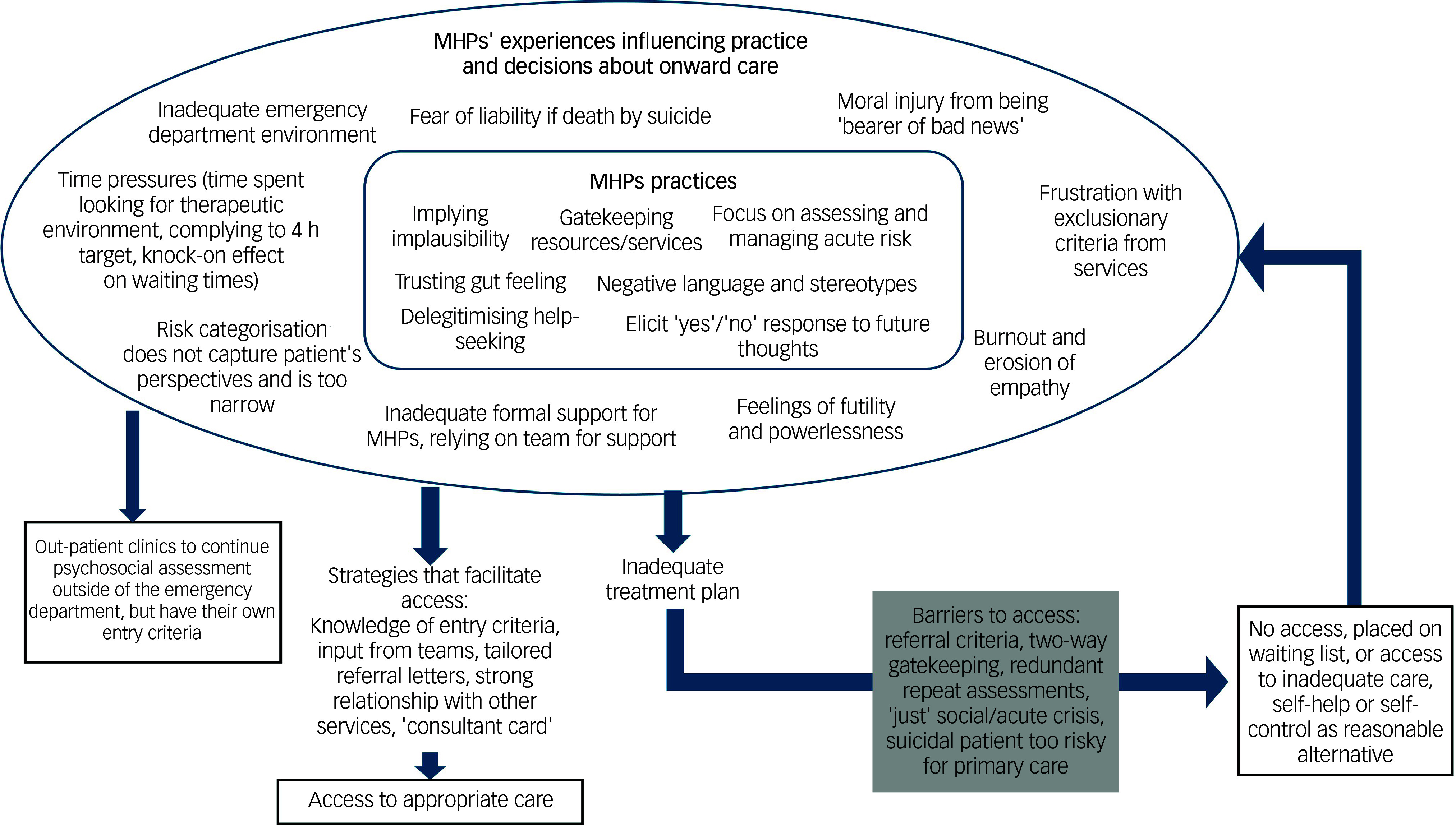
Visual model of MHP experiences and practices, and impact on decisions and patient pathways. MHP, mental health practitioner.

Figure 3 shows a visual representation of integrated findings. The model comprises three core components: MHP practices (inner rectangular box), MHP experiences that influence practice (outer oval), and patient outcomes and barriers and facilitators to access (arrows). It depicts a dynamic cycle in which MHP experiences shape practice, which influences patient trajectories perpetuating repeat emergency department attendances in a constrained and risk-focused system.

## Discussion

### Summary of main findings

This review synthesised research on MHPs’ experiences and practices in deciding onward care for patients presenting to emergency departments with self-harm or suicidal ideation. It showed how time and emergency department environmental constraints, risk-centric culture, liability fears driving defensive and gatekeeping practices, and restricted mental health services intersect to influence aftercare decisions that negatively affect patient mental health and safety.

Most studies were qualitative, with six out of 11 published after 2021, indicating a growing focus on this area, possibly accelerated by COVID-19-related pressures on emergency departments. This reflects increasing recognition of the strain placed on both acute services and the professionals operating within them.

### Comparison with existing literature

Risk is central to MHPs experiences and practices. The risk-centric culture described is pervasive in other acute care settings^30^ and echoes patient experiences of risk-focused assessments^31^ that affect shared decision-making.^32^ MHPs in our review criticised risk assessments as narrow and unreflective of the complexity of suicide risk and patient distress. Experienced MHPs adapted using informal heuristics, such as ‘gut feeling’, to capture complexity.

Despite these adaptive approaches, risk assessments remain entrenched due to their institutional function. Categorising suicide risk renders clinical uncertainty manageable, and documentation makes decisions auditable and organisationally defensible. The review identified MHPs elicit definite responses about future suicidal intent to record an audit trail to demonstrate due diligence and accountability. Such practices were previously reported by Beale in an opinion piece, who reflected: ‘It is almost as if we take the worst-case scenario and work backwards from there, starting at “this person might kill themselves”, followed by “how can I prove it wasn’t my fault?”’ (^33^, p. 16). This review underscores how these practices reflect broader structural norms of risk governance and institutional defensiveness.

While recent evidence^7,34^ and policy guidelines^4^ advocate a relational and compassionate approach to psychosocial assessments, such practices can only be ‘smuggled’ in^35^ rather than structurally supported and systemically encouraged. Entrenched liability concerns, time pressures and systemic constraints continue to drive defensive practices and reinforce a narrow, risk-centric culture at the expense of therapeutic engagement.

Signposting patients to onward care following discharge from an emergency department is a core part of an MHPs role, yet the review shows rigid referral criteria shape onward care decisions. It underlines previously reported challenges patients face in accessing psychological therapies.^36–38^ MHPs recognised the need for psychological interventions that address the underlying causes of self-harm or suicidal ideation, but felt constrained by time, lack of training and organisational expectations. As a result, care is often reduced to short-term crisis management.

MHPs adapted to these constraints through gatekeeping practices. This included reframing help-seeking as misaligned with services’ remit, emphasising self-management and coping strategies, and redirecting responsibility to the patient or to informal support networks. These practices can shape patients’ perceived candidacy, i.e. the negotiated process to access services shaped by how patients and their needs are presented, perceived and evaluated by practitioners and institutions.^39^ In this context, gatekeeping practices influence eligibility and access to care. Consistent with wider literature,^30,40^ our review shows MHPs act as adjudicators of candidacy, ensuring alignment with institutional agendas.

This review is the first, to our knowledge, to synthesise the emotional toll MHPs experience when working with patients who are suicidal in an emergency department setting. MHPs experience both the emotional intensity of patient crises and the institutional expectation that they should prevent all suicides. As one MHP reflected, ‘psychiatry is the only specialty that isn’t allowed to have a death rate’ (^21^, p. 8). Risk assessments and documentation were used not only to guide care but to protect against blame. Our findings align with previous literature highlighting MHPs experiences of coroner’s inquests as traumatising especially when clinical decisions were judged without recognition of systemic constraints.^41^

MHPs expressed a strong commitment to compassionate, trauma-informed care, yet reported negative attitudes and stigmatising language. These attitudes were described to be passed down by senior staff and embedded in workplace culture. This is reflective of staff attitudes experienced by patients in the wider mental health system.^42^

These dynamics reflect broader patterns of moral injury and compassion fatigue where practitioners are unable to act in line with their values because of structural constraints.^43^ Similar patterns have been reported by emergency department and general nursing staff, where moral distress is associated with poor mental health outcomes and workforce attrition.^44,45^ This review contributes novel insights by showing how MHPs experience moral injury when acting as gatekeepers, particularly when they deny care they think is needed.

MHPs relied on informal peer support to cope, which offered solidarity but lacked formal structure. This mirrors broader findings that peer support is valued, but insufficient to buffer against burnout.^46^ Without formal structures for debriefing or reflective supervision, the burden of managing emotional impact falls on individuals and immediate teams. As Dean et al^46^ argue, moral injury is a symptom of a broken system, not a broken individual, and addressing it requires creating environments that value time, trust and therapeutic relationships with patients.

### Strengths and limitations

To our knowledge, this is the first systematic review to synthesise MHPs’ experiences and practices for people who present to emergency departments with suicidal ideation or self-harm. It provides a comprehensive understanding of how clinical, organisational and systemic factors interact to shape MHP experiences and interactions with patients.

Only two quantitative studies were identified. Certainty of evidence from these two studies, as assessed using the GRADE approach, was very low and there was limited scope for integration between qualitative and quantitative data. Nevertheless, the design allowed meaningful thematic synthesis of qualitative data. This provided nuanced interpretation by situating MHP practices and experiences within the institutional context of the emergency department, and the overall design supported a comprehensive understanding of how MHPs make decisions and the conditions under which they occur.

All studies were conducted in high-income Western countries with established specialised liaison mental health teams, which may limit the relevance and applicability of findings to other settings. Nonetheless, the findings offer valuable insights as more emergency departments globally move toward embedding mental health services.^47^ The review also focused exclusively on MHPs’ perspectives. Although analytically purposeful, it excludes complementary insights from patients and other healthcare professionals. The inclusion of only English-language publications also introduces potential bias.

### Implications and future research

Shifting away from risk-centric and gatekeeping cultures in emergency departments requires coordinated changes in practice, training and systems. MHPs should be supported to adopt relational and compassionate approaches. This involves training and implementation of risk formulation rather than risk stratification in interaction and documentation;^7^ training that prepares MHPs to navigate uncertainty to articulate nuanced risk. Training must be grounded in real-world institutional constraints so that MHPs can manage ethical tensions and clinical ambiguity.

Reducing the emotional toll and moral distress requires regular supervision and structured peer support that explores the complexity of care and institutional constraints that pose ethical challenges. Services should foster team-based decision-making to reduce the burden of sole accountability on individual MHPs and mitigate defensive practices.

At a systems level, policies should continue to discourage risk stratification and address structural conditions that drive exclusion. Performance metrics should prioritise relational care and continuity over quick discharge and risk management, and governance structures should move toward a model of shared responsibility and institutional learning. Documentation requirements should facilitate clinical reasoning rather than reduce complex cases to defensible risk statements.

The review identified innovative models, such as out-patient clinics, to enable more comprehensive assessment and continuation of care beyond the emergency department. Other examples include mental health crisis assessment centres (or mental health emergency departments) for emergency mental health presentations that would otherwise seek treatment in emergency departments.^48^ However, only one study contributed to this finding, reducing our confidence in it, and further evaluation of such models is needed.

More high-quality quantitative studies are needed to complement existing qualitative evidence. These could clarify associations between systemic factors and outcomes such as repeat emergency department presentations, care quality and staff well-being. Research should also evaluate extended care models that address continuity and reduce fragmentation. Finally, studies should explore emergency department mental health experiences and practices in low-resource settings where service structures differ from high-income contexts.

In conclusion, MHPs are required to make decisions about onward care in chaotic and pressured emergency departments, where the complexity of suicide risk is not adequately recognised. Defensive and gatekeeping practices reflect adaptations to uncertainty, resource scarcity in the emergency department and the wider mental health system, and rigid referral criteria in secondary care. Addressing these issues requires reform across clinical, policy and research levels. Future research should focus on evaluating innovative models that recognise and support the complexity of frontline care, and reframe psychosocial assessment as a relational compassionate practice that improves patient outcomes and practitioner well-being.

## Supporting information

Suzuki et al. supplementary material 1 Suzuki et al. supplementary material

Suzuki et al. supplementary material 2 Suzuki et al. supplementary material

Suzuki et al. supplementary material 3 Suzuki et al. supplementary material

Suzuki et al. supplementary material 4 Suzuki et al. supplementary material

Suzuki et al. supplementary material 5 Suzuki et al. supplementary material

Suzuki et al. supplementary material 6 Suzuki et al. supplementary material

Suzuki et al. supplementary material 7 Suzuki et al. supplementary material

## Data Availability

Data availability is not applicable to this article as no new data were created or analysed in this study.

## Supplementary material

The supplementary material is available online at https://doi.org/10.1192/bjo.2026.11007
