# Supplementary material for: Mental health practitioners’ experiences and practices in making decisions about onward care for patients presenting to emergency departments with self-harm or suicidal ideation: systematic review and meta-synthesis
Source: BJPsych Open. 2026 Mar 30;12(3):e95. doi: 10.1192/bjo.2026.11007 (PMC13107293; doi:10.1192/bjo.2026.11007)
Supplement: Suzuki et al. supplementary material 1 — Suzuki et al. supplementary material [file S2056472426110072sup001.docx]

**MEDLINE**

(self adj1 (harm* or inju* or mutilat* or destruct* or poison* or immolat* or incinerat* or inflict* or lacerat* or cut*)).ti,ab,kf.
((themselves or "their self") adj3 (harm* or mutilat* or destruct* or posison* or cut*)).ti,ab,kf.
(Suicid$ or auto-mutilat* or auto?mutilat* or overdos* or fatal behavio?r).ti,ab,kf.
(suicid* adj1 (ideat* or thought*)).ti,ab,kf.
exp suicide/
or/1-5
((liaison or mental health or psychia* or psychol*) adj2 (worker* or practitioner* or provider* or manager* or team* or service* or profession* or clinician* or doctor* or medic* staff or nurs* or physician*)).ti,ab,kf.
(psychiatrist* or therapist? or social worker? or psycholog* or occupational health? or care?coordinator* or MHP).ti,ab,kf.
or/7-8
(emergency adj2 (hospital* or department or medicine or service or medical or room)).ti,ab,kf.
(accident?emergency or ER or ED or A?E or hospital*).ti,ab,kf.
emergency department/
or/10-12
(attitud* or belie* or cultur* or perspectiv* or stereotyp* or knowledg* or stigma* or opinion* or view* or disposition* or feel* or impression* or characteri* or experien*).ti,ab,kf.
attitude/
(decision* or process or judg?ment* or assessment* or management* or care* or plan* or "safety plan*" or formulat* or clinical judg* or "shared decision?making*" or "person?cent*" or treatment* or discharg* or refer* or communicat*).ti,ab,kf.
exp Clinical Decision-Making/
Decision Making/
or/14-15
or/16-18
or/14-18
6 and 9 and 13 and 19 and 20
6 and 9 and 13 and 20
6 and 9 and 13 and 21

**EMBASE/ EMCARE**

(self adj1 (harm* or inju* or mutilat* or destruct* or poison* or immolat* or incinerat* or inflict* or lacerat* or cut*)).ti,ab,kf.
((themselves or "their self") adj3 (harm* or mutilat* or destruct* or posison* or cut*)).ti,ab,kf.
(Suicid$ or auto-mutilat* or auto?mutilat* or overdos* or fatal behavio?r).ti,ab,kf.
(suicid* adj1 (ideat* or thought*)).ti,ab,kf.
Suicide/

Suicidal ideation/

Suicidal behavior/

Suicide attempt/

Suicide prevention/

Automutilation/

OR/1-10

((liaison or mental health or psychia* or psychol*) adj2 (worker* or practitioner* or provider* or manager* or team* or service* or profession* or clinician* or doctor* or medic* staff or nurs* or physician*)).ti,ab,kf.
(psychiatrist* or therapist? or social worker? or psycholog* or occupational health? or care?coordinator* or MHP).ti,ab,kf.
OR/12-13

(emergency adj2 (care or hospital* or department or medicine or service or medical or room)).ti,ab,kf.
(accident?emergency or ER or ED or A?E or hospital*).ti,ab,kf.
emergency ward/

OR/15-17

(attitud* or belie* or cultur* or perspectiv* or stereotyp* or knowledg* or stigma* or opinion* or view* or disposition* or feel* or impression* or characteri* or experien*).ti,ab,kf.
 “health personnel attitude”/

OR/18-19

(decision* or process or judg?ment* or assessment* or management* or care* or plan* or "safety plan*" or formulat* or clinical judg* or "shared decision?making*" or "person?cent*" or treatment* or discharg* or refer* or communicat*).ti,ab,kf.
clinical decision making/

clinical reasoning/

shared decision making/

patient decision making/

medical decision making/

OR/21-26

**PSYCINFO – EBSCOhost**

TI ( (self n1 (harm* or inju* or mutilat* or destruct* or poison* or immolat* or incinerat* or inflict* or lacerat* or cut*)) ) OR AB ( (self n1 (harm* or inju* or mutilat* or destruct* or poison* or immolat* or incinerat* or inflict* or lacerat* or cut*)) ) OR KW (self n1 (harm* or inju* or mutilat* or destruct* or poison* or immolat* or incinerat* or inflict* or lacerat* or cut*))

TI ((themselves or "their self") n3 (harm* or mutilat* or destruct* or posison* or cut*)) OR AB ((themselves or "their self") n3 (harm* or mutilat* or destruct* or posison* or cut*)) OR KW ((themselves or "their self") n3 (harm* or mutilat* or destruct* or posison* or cut*))

TI (Suicid$ or auto-mutilat* or auto?mutilat* or overdos* or fatal behavio?r) OR AB (Suicid$ or auto-mutilat* or auto?mutilat* or overdos* or fatal behavio?r) OR KW (Suicid$ or auto-mutilat* or auto?mutilat* or overdos* or fatal behavio?r)

TI (suicid* n1 (ideat* or thought*)) OR AB (suicid* n1 (ideat* or thought*)) OR KW (suicid* n1 (ideat* or thought*))

DE “suicidal behavior+”

DE”suicide prevention”

DE "Nonsuicidal Self-Injury"

DE"suicidality"

TI ((liaison or mental health or psychia* or psychol*) n2 (worker* or practitioner* or provider* or manager* or team* or service* or profession* or clinician* or doctor* or medic* staff or nurs* or physician*)) OR AB ((liaison or mental health or psychia* or psychol*) n2 (worker* or practitioner* or provider* or manager* or team* or service* or profession* or clinician* or doctor* or medic* staff or nurs* or physician*)) OR KW ((liaison or mental health or psychia* or psychol*) n2 (worker* or practitioner* or provider* or manager* or team* or service* or profession* or clinician* or doctor* or medic* staff or nurs* or physician*))

TI (psychiatrist* or therapist? or social worker? or psycholog* or occupational health? or care?coordinator* or MHP) OR AB (psychiatrist* or therapist? or social worker? or psycholog* or occupational health? or care?coordinator* or MHP) OR KW (psychiatrist* or therapist? or social worker? or psycholog* or occupational health? or care?coordinator* or MHP)

TI (emergency n2 (hospital* or department or medicine or service or medical or room)) OR AB (emergency n2 (hospital* or department or medicine or service or medical or room)) OR KW (emergency n2 (hospital* or department or medicine or service or medical or room))

TI (accident?emergency or ER or ED or A?E or hospital*) OR AB (accident?emergency or ER or ED or A?E or hospital*) OR KW (accident?emergency or ER or ED or A?E or hospital*)

DE “emergency services"

TI (attitud* or belie* or cultur* or perspectiv* or stereotyp* or knowledg* or stigma* or opinion* or view* or disposition* or feel* or impression* or characteri* or experien*) OR AB (attitud* or belie* or cultur* or perspectiv* or stereotyp* or knowledg* or stigma* or opinion* or view* or disposition* or feel* or impression* or characteri* or experien*) or KW (attitud* or belie* or cultur* or perspectiv* or stereotyp* or knowledg* or stigma* or opinion* or view* or disposition* or feel* or impression* or characteri* or experien*)
DE "health personnel attitudes"

TI (decision* or process or judg?ment* or assessment* or management* or care* or plan* or "safety plan*" or formulat* or clinical judg* or "shared decision?making*" or "person?cent*" or treatment* or discharg* or refer* or communicat*) OR AB (decision* or process or judg?ment* or assessment* or management* or care* or plan* or "safety plan*" or formulat* or clinical judg* or "shared decision?making*" or "person?cent*" or treatment* or discharg* or refer* or communicat*) or KW (decision* or process or judg?ment* or assessment* or management* or care* or plan* or "safety plan*" or formulat* or clinical judg* or "shared decision?making*" or "person?cent*" or treatment* or discharg* or refer* or communicat*)

DE "clinical judgment (not diagnosis)"

DE "decision making"

S1 OR S2 OR S3 OR S4 OR S5 OR S6 OR S7 OR S8 - suicide

S9 OR S10 - liaison

S11 OR S12 OR S13 – emergency

S14 OR S15 – attitude

S16 OR S17 OR S18 – decision

**CINAHL**

TI ( (self n1 (harm* or inju* or mutilat* or destruct* or poison* or immolat* or incinerat* or inflict* or lacerat* or cut*)) ) OR AB ( (self n1 (harm* or inju* or mutilat* or destruct* or poison* or immolat* or incinerat* or inflict* or lacerat* or cut*)) )

TI ((themselves or "their self") n3 (harm* or mutilat* or destruct* or posison* or cut*)) OR AB ((themselves or "their self") n3 (harm* or mutilat* or destruct* or posison* or cut*))

TI (Suicid$ or auto-mutilat* or auto?mutilat* or overdos* or fatal behavio?r) OR AB (Suicid$ or auto-mutilat* or auto?mutilat* or overdos* or fatal behavio?r)

TI (suicid* n1 (ideat* or thought*)) OR AB (suicid* n1 (ideat* or thought*))

(MH "Suicide+")

(MH "Self-Injurious Behavior")

TI ((liaison or mental health or psychia* or psychol*) n2 (worker* or practitioner* or provider* or manager* or team* or service* or profession* or clinician* or doctor* or medic* staff or nurs* or physician*)) OR AB ((liaison or mental health or psychia* or psychol*) n2 (worker* or practitioner* or provider* or manager* or team* or service* or profession* or clinician* or doctor* or medic* staff or nurs* or physician*))

TI (psychiatrist* or therapist? or social worker? or psycholog* or occupational health? or care?coordinator* or MHP) OR AB (psychiatrist* or therapist? or social worker? or psycholog* or occupational health? or care?coordinator* or MHP)

TI (emergency n2 (hospital* or department or medicine or service or medical or room)) OR AB (emergency n2 (hospital* or department or medicine or service or medical or room))

TI (accident?emergency or "ER" or "ED" or "A?E" or hospital*) OR AB (accident?emergency or "ER" or "ED" or "A?E" or hospital*)

MH "emergency service"

TI (attitud* or belie* or cultur* or perspectiv* or stereotyp* or knowledg* or stigma* or opinion* or view* or disposition* or feel* or impression* or characteri* or experien*) OR AB (attitud* or belie* or cultur* or perspectiv* or stereotyp* or knowledg* or stigma* or opinion* or view* or disposition* or feel* or impression* or characteri* or experien*)

MH "attitude of health personnel"

TI (decision* or process or judg?ment* or assessment* or management* or care* or plan* or "safety plan*" or formulat* or clinical judg* or "shared decision?making*" or "person?cent*" or treatment* or discharg* or refer* or communicat*) OR AB (decision* or process or judg?ment* or assessment* or management* or care* or plan* or "safety plan*" or formulat* or clinical judg* or "shared decision?making*" or "person?cent*" or treatment* or discharg* or refer* or communicat*)

MH "Decision Making, Clinical+"

MH "Decision Making, Shared"

MH "Decision Making"

**Web of Science**

TS=(self near/1 (harm* or inju* or mutilat* or destruct* or poison* or immolat* or incinerat* or inflict* or lacerat* or cut*))
TS= ((themselves or "their self") near/3 (harm* or mutilat* or destruct* or posison* or cut*))
TS= (Suicid$ or auto-mutilat* or auto?mutilat* or overdos* or fatal behavio?r)
TS= (suicid* near/1 (ideat* or thought*))

TS= ((liaison or mental health or psychia* or psychol*) near/2 (worker* or practitioner* or provider* or manager* or team* or service* or profession* or clinician* or doctor* or medic* or staff or nurs* or physician*))
TS= (psychiatrist* or therapist? or social worker? or psycholog* or occupational health? or care?coordinator* or MHP)

TS= (emergency near/2 (hospital* or department or medicine or service or medical or room))
TS= (accident?emergency or ER or ED or A?E or hospital*)

TS= (attitud* or belie* or cultur* or perspectiv* or stereotyp* or knowledg* or stigma* or opinion* or view* or disposition* or feel* or impression* or characteri* or experien*)

TS= (decision* or process or judg?ment* or assessment* or management* or care* or plan* or "safety plan*" or formulat* or clinical judg* or "shared decision?making*" or "person?cent*" or treatment* or discharg* or refer* or communicat*)
