# Supplementary material for: Mental health practitioners’ experiences and practices in making decisions about onward care for patients presenting to emergency departments with self-harm or suicidal ideation: systematic review and meta-synthesis
Source: BJPsych Open. 2026 Mar 30;12(3):e95. doi: 10.1192/bjo.2026.11007 (PMC13107293; doi:10.1192/bjo.2026.11007)
Supplement: Suzuki et al. supplementary material 2 — Suzuki et al. supplementary material [file S2056472426110072sup002.docx]

**Supplementary materials 2 – MMAT quality assessment**

Qualitative quality assessment using MMAT

|  | Appropriate qualitative approach | Adequate data collection methods | Findings derived from the data | Interpretation substantiated | Coherence between data, collection, analysis and interpretation | Comment for ‘Can’t tell’ or ‘X’ |
| --- | --- | --- | --- | --- | --- | --- |
| Chunduri et al.,2019 | ✓ | ✓ | ✓ | ✓ | ✓ |  |
| Rheinberger et al., 2022 | ✓ | ✓ | ✓ | ✓ | ✓ |  |
| Murphy et al., 2019 | ✓ | ✓ | ✓ | ✓ | ✓ |  |
| McCarthy et al., 2023 | ✓ | ✓ | ✓ | ✓ | ✓ |  |
| Quinlivan et al., 2023 | ✓ | ✓ | ✓ | ✓ | ✓ |  |
| O’Keeffe et al., 2021 | ✓ | ✓ | ✓ | ✓ | ✓ |  |
| Bergen, Bortolloti, et al., 2023 | ✓ | ✓ | ✓ | ✓ | ✓ |  |
| Bergen, Lomas, et al., 2023 | ✓ | ✓ | ✓ | ✓ | ✓ |  |
| McCabe et al., 2023 | ✓ | ✓ | ✓ | ✓ | ✓ |  |
| Phillips et al., 2015 (Qualitative) | ✓ | ✓ | Can’t tell | X | Can’t tell | Weak analytic depth, insufficient substantiation of interpretation; lack of transparency on coherence |

Quantitative (descriptive) quality assessment using MMAT

|  | Relevant sampling strategy | Representative sample | Appropriate measurements | Low nonresponse bias | Appropriate statistical analysis | Comment for ‘Can’t tell’ or ‘X’ |
| --- | --- | --- | --- | --- | --- | --- |
| Haslam & Jones, 2020 | ✓ | X | X | Can’t tell | X | Lack of a representative sample; lacked transparency on statistical analyses. |
| Phillips et al., 2015 (Quantitative) | ✓ | X | ✓ | X | ✓ | non-representative sample, low response rate (9.59%) affecting nonresponse bias. |

Mixed-method quality assessment using MMAT

|  | Clear rationale for using mixed methods | Effective integration of components | Addressed divergences or inconsistencies | Adequate consideration of integration limitations | Quality criteria for each method met | Comment for ‘Can’t tell’ or ‘X’ |
| --- | --- | --- | --- | --- | --- | --- |
| Phillips et al., 2015 (Mixed method) | ✓ | X | ✓ | X | X | Integration of findings was limited; poor quality of each component. |
