# Supplementary material for: Mental health practitioners’ experiences and practices in making decisions about onward care for patients presenting to emergency departments with self-harm or suicidal ideation: systematic review and meta-synthesis
Source: BJPsych Open. 2026 Mar 30;12(3):e95. doi: 10.1192/bjo.2026.11007 (PMC13107293; doi:10.1192/bjo.2026.11007)
Supplement: Suzuki et al. supplementary material 3 — Suzuki et al. supplementary material [file S2056472426110072sup003.docx]

Supplementary Material 3: Evidence Profile of Quantitative Review Findings

**Quantitative review finding 1:** There is substantial variability in MHP admission decisions (ranging from 58% to 96%) even when intent is judged similarly, suggesting that social factors like isolation or support influence decisions as much as clinical risk.

Population: Mental health practitioners

Context: Decisions following self-harm

Outcome: Admission versus non-admission decisions

Contributing studies: Phillips et al., 2015

| **Risk of bias** | **Inconsistency** | **Indirectness** | **Imprecision** | **Publication bias** | **Overall certainty of evidence** | **Explanation of overall assessment** |
| --- | --- | --- | --- | --- | --- | --- |
| Serious concerns: Poor quality of evidence with some methodological concerns | Not applicable:  Single study. Cannot assess consistency across studies. | Serious concern: Despite rigorous validation of vignettes, these are a proxy and does not fully capture the complexity of real ED decision-making. | Serious concern: only one study contributing to review finding resulting in small overall sample size (n=211). | Not applicable:  Single study; no indication of selective reporting, but assessment limited. | Very low (⊕◯◯◯) | Downgraded for risk of bias, indirectness, imprecision. Could not assess inconsistency or publication bias as only one study contributed to finding. |

**Quantitative review finding 2:** There was no significant association between breach of 4-hour time target before referral to mental health liaison team on whether patient was discharged (*X*^2^(1) =0.091=*p*<0.763).

Population: Patients referred to liaison mental health services

Context: Emergency department

Outcome: Discharge from hospital (yes/no)

Contributing studies: Haslam & Jones, 2019

| **Risk of bias** | **Inconsistency** | **Indirectness** | **Imprecision** | **Publication bias** | **Overall certainty of evidence** | **Explanation of overall assessment** |
| --- | --- | --- | --- | --- | --- | --- |
| Serious concerns: Potential confounding by case complexity, staffing levels, and service availability not adjusted for. | Not applicable:  Single study. Cannot assess consistency across studies. | Serious concern: Referral pathway used as a proxy for decision-making; clinical rationale for referrals not directly examined. | Serious concern: only one study contributing to review finding resulting in small overall sample size (n=734 episodes). | Not applicable:  Single study; no indication of selective reporting, but assessment limited. | Very low (⊕◯◯◯) | Downgraded for risk of bias, indirectness, imprecision. Could not assess inconsistency or publication bias as only one study contributed to finding. |

**Quantitative review finding 3:** Patients seen within 4-hour target were more often referred for Mental Health Act assessment, while those seen after 4-hour target breached were more likely referred to a CDU, a short-stay unit where a patient receives further observation, assessment or support.

Population: Patients referred to liaison mental health services

Context: Emergency department

Outcome: Referral pathway among non-discharged patients (psychiatric admission, Mental Health Act assessment, crisis decision unit)

Contributing studies: Haslam & Jones, 2019

| **Risk of bias** | **Inconsistency** | **Indirectness** | **Imprecision** | **Publication bias** | **Overall certainty of evidence** | **Explanation of overall assessment** |
| --- | --- | --- | --- | --- | --- | --- |
| Serious concerns: Potential confounding by case complexity, staffing levels, and service availability not adjusted for. | Not applicable:  Single study. Cannot assess consistency across studies. | Serious concern: Referral pathway used as a proxy for decision-making; clinical rationale for referrals not directly examined. | Serious concern: only one study contributing to review finding resulting in small overall sample size (n=734 episodes). | Not applicable:  Single study; no indication of selective reporting, but assessment limited. | Very low (⊕◯◯◯) | Downgraded for risk of bias, indirectness, imprecision. Could not assess inconsistency or publication bias as only one study contributed to finding. |
