# Supplementary material for: Mental health practitioners’ experiences and practices in making decisions about onward care for patients presenting to emergency departments with self-harm or suicidal ideation: systematic review and meta-synthesis
Source: BJPsych Open. 2026 Mar 30;12(3):e95. doi: 10.1192/bjo.2026.11007 (PMC13107293; doi:10.1192/bjo.2026.11007)
Supplement: Suzuki et al. supplementary material 5 — Suzuki et al. supplementary material [file S2056472426110072sup005.docx]

Table 1. Summary of included studies

| Author, Year | Country | Study  design | Study aim(s) | Description of ED MHPs are based | (Sample size)  Description of practitioner roles | Practitioner Age | % Female | Description of patient population | Main findings |
| --- | --- | --- | --- | --- | --- | --- | --- | --- | --- |
| Qualitative studies | | | | | | | | | |
| Bergen, Bortolloti, et al., 2023 | UK | Conversation analysis | To identify communication practices used to either undermine, imply/assert alternative characterizations or accept and validate peoples’ accounts of self-harm and suicidal ideation | Did not report | N=5 MHP  Mental health nurse (N=2), Occupational therapist (N=2), Social worker (N=1) | Range: 40-60 | 60% | Self-harm and/or  suicidal ideation | - Communication practices that “implied implausibility”, i.e. questioned the credibility of patients’ self-harm and suicidal ideation, undermining their experiences |
| Bergen, Lomas, et al., 2023 | UK | Conversation analysis | To identify what rationales are systematically used in decisions not to refer to specialist mental health services after a patient request for support | Did not report | (N=46 video recordings from N= 33 MHP)    Mental health nurses (N = 11), a student nurse (N = 1), doctors (N = 2), junior doctors (N = 7), social workers (N = 2), occupational therapists (N = 2), healthcare assistants (N = 2), consultant psychiatrists (N = 2), psychiatry trainees (N = 2) and unspecified (N = 2) | Did not report | 61% | Self-harm and/or  suicidal ideation | - Communication practice where MHPs acted as gatekeepers to onward care when patients asked for more support |
| McCarthy et al., 2023 | UK | Semi-structured interviews | To explore and synthesise the perspectives of ED administrative, medical and mental health staff working with people presenting in suicidal crisis to gain novel insights into the key challenges ED staff face when providing care in UK ED settings | Six EDs:  Three EDs  were in urban areas.  Three in rural areas. Two Mental  Health Trusts were covered by the ED, which are responsible for providing  health and social care services for people with mental health dis-  orders. | (N=23)    ED receptionist (N=2), Physician associate (N=1), Triage nurse (N=1), Practice development nurse (N=1), ED doctor (N=2), Consultant in emergency medicine (N=4), Mental health nurse (N=4), mental health nursing student (N=2), Liaison psychiatry doctor (N=1), Consultant liaison psychiatrist (N=2), Crisis team manager (n=1), Advance nurse practitioner (N=2) | Did not report | 57% | Self-harm and/or  suicidal ideation | - Emotional exhaustion and burnout - Defensive practice was common - Focus on risk and liability - Increased training in psychological therapies - Want more support for MHPs |
| Chunduri et al., 2019 | USA | Focus groups | To explore suicide risk identification and flow of patients with differing suicide risk through the Psychiatric Emergency Service (PES) to their clinical dispositions. | Large  public teaching hospital | (N=15)  Psychiatrists  Physician assistant  Nurse practitioner; Sample breakdown not reported | Did not report | Did not report | Self-harm and/or suicidal ideation – suicide risk | - Primary focus on supporting acute presentations (not chronic) - Suspect malingering in patients with positive risk assessment and search for inconsistencies - Experience is important for risk assessments - Frustration with documentation but helpful to have narrative notes for assessment - Concerns about legal liability of adverse events - Unrealistic expectation to prevent all suicides - Multidisciplinary teams help with assessments but responsibility is ultimately on the individual - Learn by seeing patient outcomes |
| Murphy et al., 2019 | Ireland | Interviews – qualitative descriptive design | To explore how mental health nurses understand and work with repeated self-harm | Did not report | (N=9)  Clinical Nurse specialist: Liaison Mental Health Nurse, A&E department (N=1), Staff nurse: Female psychiatric ICU (N=1), Staff nurse: Community Day Hospital (N=1), Staff nurse: Female psychiatric ICU (N=1), Staff nurse: Acute Inpatient Unit (N=1), Community mental health nurse (N=1), Self-harm nurse in A&E department (N=1), Community mental health nurse (N=1), Staff nurse: Acute Inpatient Unit (N=1) | Did not report | 89% | Repeat self-harm | - MHPs understand self-harm as a coping mechanism, not just a behaviour to be stopped immediately - MHP training can combat negative stereotypes |
| Quinlivan et al., 2023 | UK | Qualitative multi-site interview study | To explore barriers and facilitators to accessing aftercare and psychological therapies for patients presenting to hospital following self-harm, from the perspective of liaison psychiatry practitioners | 32 randomly selected hospital sites from around England.  The sample was stratified to  include small and large hospitals with high and low admission  rates for self-harm. | (N=51)  Consultant liaison psychiatrists (N=13), Mental health nurse (N=22), Team managers/clinical leads (N=11), Psychologist/psychotherapist (N=4), higher specialty trainee doctor (N=1) | Did not report | 57% | Self-harm only | - Barriers to accessing services due to restrictive referral criteria and long waiting times - Cycle where patients with unmet needs returned repeatedly to the ED - Strategies to facilitate referrals included tailoring referral letters and leveraging relationships with other services |
| McCabe et al., 2023 | UK | Conversation analysis - Cross-sectional non-participant observational study of clinical practice | To micro-analyse how clinicians ask about suicidal ideation and/ or self-harm in emergency department psychosocial assessments and how patients respond | Did not report | (N=46 video recordings from N= 33 MHP) 23 MHP included in analysis    Mental health nurses (N = 11), a student nurse (N = 1), doctors (N = 2), junior doctors (N = 7), social workers (N = 2), occupational therapists (N = 2), healthcare assistants (N = 2), consultant psychiatrists (N = 2), psychiatry trainees (N = 2) and unspecified (N = 2) | Mean: 38.3  Range: 23-62 | 61% | Self-harm only | - Open-ended questions more effective in encouraging patient disclosure of self-harm compared to closed questions with negative phrasing (65% vs. 8%) |
| O’Keeffe et al., 2021 | UK | Focus group and interviews | To explore treatment of self-harm in emergency departments, comparing perspectives of patients, carers and practitioners | Four EDs across  London and the South West of England. | (N= 37)  Psychiatrists (N=4), Junior doctors (N=11), Nurses (N= 18, 49%), Psychologist (N=3, 8%), Occupational therapists (N=1, 3%) | Mean: 37  Range: 21-63 | 73% | Self-harm only | - ED environment lack of privacy and time constraints, which impacted their ability to conduct thorough assessments - Defensive practice was common - Focus on risk and liability - Increased training in psychological therapies |
| Rheinberger et al., 2022 | Australia | Focus groups and interviews | To explore the experience of providing care to individuals in a suicide crisis from the perspectives of staff in a variety of roles within the ED to gain novel insights into key challenges preventing them from offering adequate care to individuals experiencing a suicide crisis | Two EDs in large metropolitan area ED1 was part of the local public  hospital and had approximately 87,000 ED presentations.  ED2 was part of a large private hospital and saw approximately 55,500 ED presentations. | (N = 54)    ED nurses (N=13), Registrars (N=11), Senior Resident Medical Officer (N=7), Specialists (N=18), Mental health nurse (N=2), Psychiatric registrar (N=1)  Note: only findings from mental health nurses and psychiatric registrar were included in analysis | Did not report | 57%  (Sex of 5 participants not reported) | Self-harm and/or suicidal ideation | - Frustration with standardized risk assessments - Sometimes relied on intuition ("gut feeling") in decision-making - Difficult to stratify patients who fell between high- and low-risk categories - The chaotic ED environment - Emotional exhaustion, burnout, and frustration due to systemic barriers - Lack of adequate support for MHPs |
| Quantitative study | | | | | | | | | |
| Haslam & Jones, 2020 | UK | Retrospective case-control | To explore the impact of the four-hour target upon the discharge  decisions for those patients who have self-harmed; exploring the potential relationship between the  decision to discharge from ED, and whether the patient first breached the four-hour target | Two EDs offering 24-h service | (N = 14)    Senior mental health nurses (N =10), Health care assistants (N =4) | Did not report | Did not report | Self-harm only | - No significant difference in admission rates based on time targets - Patients seen within the 4-hour target were more likely to be referred for mental health act assessments |
| Mixed-methods study | | | | | | | | | |
| Phillips et al., 2015 | Australia | Survey response to vignettes and open-response text | To explore variations in patient disposition decisions, following risk assessment for deliberate self-harm by mental health nurses in the ED setting | Did not report | (N=211)    Did not report specifics of professional roles but sample pool was nurses | Did not report | 68.6% | Self-harm only (“Deliberate  Self-harm”) | - 96% agreement with suicidal intent among practitioners - Variability in referral decisions (community-based treatment or hospitalisation) - especially with ambiguous cases MHPs made more conservative choices, e.g. opting for hospitalization - Protective factors such as social/ family support prioritised over suicidal intent |
