# Supplementary material for: Mental health practitioners’ experiences and practices in making decisions about onward care for patients presenting to emergency departments with self-harm or suicidal ideation: systematic review and meta-synthesis
Source: BJPsych Open. 2026 Mar 30;12(3):e95. doi: 10.1192/bjo.2026.11007 (PMC13107293; doi:10.1192/bjo.2026.11007)
Supplement: Suzuki et al. supplementary material 6 — Suzuki et al. supplementary material [file S2056472426110072sup006.docx]

Table 2. Summary of quantitative findings with GRADE assessment*

|  | **Number of participants (studies)** | **Certainty of evidence** | **Explanation of overall assessment** |
| --- | --- | --- | --- |
| **Quantitative review finding 1:** There is substantial variability in MHP admission decisions (ranging from 58% to 96%) even when intent is judged similarly, suggesting that social factors like isolation or support influence decisions as much as clinical risk.  Population: Mental health practitioners  Context: Decisions following self-harm  Outcome: Admission versus non-admission decisions | N=211  (1 study) | Very low | Downgraded for risk of bias, indirectness, imprecision. Could not assess inconsistency or publication bias as only one study contributed to finding. |
| **Quantitative review finding 2:** There was no significant association between breach of 4-hour time target before referral to mental health liaison team on whether patient was discharged (*X*^2^(1) =0.091=*p*<0.763).  Population: Patients referred to liaison mental health services  Context: Emergency department  Outcome: Discharge from hospital (yes/no) | N=734 episodes  (1 study) | Very low | Downgraded for risk of bias, indirectness, imprecision. Could not assess inconsistency or publication bias as only one study contributed to finding. |
| **Quantitative review finding 3:** Patients seen within 4-hour target were more often referred for Mental Health Act assessment, while those seen after 4-hour target breached were more likely referred to a CDU, a short-stay unit where a patient receives further observation, assessment or support.  Population: Patients referred to liaison mental health services  Context: Emergency department  Outcome: Referral pathway among non-discharged patients (psychiatric admission, Mental Health Act assessment, crisis decision unit) | N=734 episodes  (1 study) | Very low | Downgraded for risk of bias, indirectness, imprecision. Could not assess inconsistency or publication bias as only one study contributed to finding. |

*For further details, see Supplementary Materials 3
