# Supplementary material for: Mental health practitioners’ experiences and practices in making decisions about onward care for patients presenting to emergency departments with self-harm or suicidal ideation: systematic review and meta-synthesis
Source: BJPsych Open. 2026 Mar 30;12(3):e95. doi: 10.1192/bjo.2026.11007 (PMC13107293; doi:10.1192/bjo.2026.11007)
Supplement: Suzuki et al. supplementary material 7 — Suzuki et al. supplementary material [file S2056472426110072sup007.docx]

Table 3. Summary of qualitative review findings and GRADE-CERQual Assessment*

| **ID** | **Summary of qualitative review findings** | **Contributing qualitative studies** | **GRADE‐CERQual assessment of confidence in the evidence** | **Explanation of GRADE-CERQual assessment** |
| --- | --- | --- | --- | --- |
| **Theme 1: Risk-centric culture is anti-therapeutic and shapes defensive practice, scepticism towards patients, and burnout** | | | | |
| 1 | **Dominance of suicide risk assessment over therapeutic engagement.**  MHPs described the psychosocial assessment as being primarily oriented toward identifying and managing suicide risk. MHPs reported discomfort that risk was narrowly defined and did not adequately reflect what patients themselves experienced as most concerning. This dominant focus on risk was described as overshadowing the therapeutic potential of the assessment interaction with MHPs noting missed opportunities for therapeutic engagement. | Chunduri et al.; O’Keeffe et al.; Rheinberger et al. | High confidence | No or very minor concerns on methodological limitations, no or very minor concerns on coherence, no or very minor concerns on adequacy, minor concerns on relevance (only high-income Western context represented) |
| 2 | **Scepticism and interrogative practices when risk accounts diverge.**  When discrepancies were perceived between the patient’s account and MHP’s impressions, MHPs adopted a sceptical approach and interrogated patient’s credibility and intent by probing for inconsistencies, emphasising future orientation, or reframing suicidal behaviour as impulsive. | Chunduri et al.; Bergen Bortolotti et al. | Moderate confidence | No or very minor concerns on methodological limitations, minor concerns on adequacy (only 2 studies contributed with relatively thick data), minor concerns on coherence (risk assessment screens extrapolated to MHP’s impressions for one study), minor concerns on relevance (only high-income Western context represented) |
| 3 | **MHPs felt anxious, weary and fearful of in suicide risk assessment reliance on ‘gut feeling’.**  MHPs described suicide risk assessment as complex and uncertain, reporting feelings of anxiety, weariness and fear when making decisions about onward care in the absence of clear indicators of risk. Adhering to assessment protocols provided reassurance that professional responsibilities had been met. Some MHPs also reported drawing on clinical experience or “gut feeling” to guide decision-making with experience perceived to improve confidence and accuracy of assessments. | Chunduri et al.; McCarthy et al.; Rheinberger et al. | High confidence | No or very minor concerns on methodological limitations, no or very minor concerns on adequacy, minor concerns on coherence (one study did not explicitly link greater experience to more confidence in risk assessment but finding reflects variation), minor concerns on relevance (only high-income Western context represented) |
| 4 | **Fear of liability underlie defensive practices**  MHPs described fear of being held liable for patient suicide. This underpinned defensive practices such as narrowing the domain of risk (i.e. only considering acute risk), prioritising extensive documentation, using constrained questioning to elicit definitive answers, and shifting responsibility across service boundaries in order to demonstrate due diligence and reduce exposure to blame. Some MHPs acknowledged that this defensive posture could contribute to negative attitudes towards patients. | Chunduri et al.; McCabe et al.; McCarthy et al.; O’Keeffe et al. | High confidence | No or very minor concerns on methodological limitations, no or very minor concerns on coherence, no or very minor concerns on adequacy, minor concerns on relevance (only high-income Western context represented) |
| **Theme 2: Time and environmental pressures impact on the quality and therapeutic potential of assessments** | | | | |
| 5 | **Unsuitability of the ED physical environment for comprehensive psychosocial assessment.**  The physical environment of the ED was unsuitable for conducting thorough psychosocial assessments. MHPs reported that busy, chaotic settings and lack of privacy made it difficult to engage patient in open and thorough conversations, often requiring them to delay assessments until private space could be found or to improvise within limited facilities. These environmental constraints shaped how assessments were conducted rather than prevent them entirely. | McCarthy, et al.; O’Keeffe et al.; Rheinberger et al. | High confidence | No or very minor concerns on methodological limitations, no or very minor concerns on coherence, no or very minor concerns on adequacy, minor concerns on relevance (only high-income Western context represented) |
| 6 | **Time pressure and competing demands constraining quality of psychosocial assessment.**  MHPs experienced significant time pressure when conducting psychosocial assessments in emergency departments, linked to institutional demands to maintain patient flow and meet discharge targets. MHPs reported that limited time constrained the quality of assessments, particularly for complex presentations, and created a tension between spending adequate time with individual patients and managing long waits for others. Extensive documentation requirements were described as adding to these pressures; while some practitioners viewed documentation as burdensome and detracting from patient contact, others emphasised the importance of detailed narrative notes to communicate clinical reasoning and support continuity of care. | Chunduri et al.; McCarthy et al.; Rheinberger et al. | High confidence | No or very minor concerns on methodological limitations, no or very minor concerns on coherence, no or very minor concerns on adequacy, minor concerns on relevance (only high-income Western context represented) |
| **Theme 3: “Battling” to access services: gatekeeping, cycles of repeat attendances and moral injury** | | | | |
| 7 | **Restrictive service thresholds and categorisation limiting access to care.**  MHPs described restrictive eligibility criteria for primary and secondary mental health services. MHPs reported access to secondary care was typically limited to people with severe or enduring mental illness or high levels of assessed need, and that patients without formal psychiatric diagnoses were often excluded. Crises were described as informally categorised as “psychiatric,” “social,” or “relationship-based,” with only some categories treated as appropriate for specialist mental health input, while acute or situational crises were deprioritised under assumptions that they would resolve without further support. MHPs worried that this reinforces a situation where people need to continue to self-harm before gaining access. | Bergen, Lomas et al.; O'Keeffe et al.; Quinlivan et al. | High confidence | No or very minor concerns on methodological limitations, no or very minor concerns on coherence, no or very minor concerns on adequacy, minor concerns on relevance (UK context only) |
| 8 | **Frustration with redundancy of repeated assessments and unpredictable referral outcomes.**  MHPs described frustration with assessments being repeated by other services and referrals being rejected, both of which delayed patients’ access to care and undermined their assessment. MHPs reported that referral acceptance varied unpredictably depending on which service or clinician was contacted, reflecting inconsistent thresholds and practices across services. | Chunduri et al; Quinlivan et al. | High confidence | No or very minor concerns on methodological limitations, no or very minor concerns on coherence, minor concerns on adequacy (only 2 studies contributed relatively thick data), minor concerns on relevance (only high-income Western context represented) |
| 9 | **Gatekeeping and moral distress under resource constraints.**  MHPs described being positioned as gatekeepers to scarce mental health resources, such as inpatient beds. This gatekeeping was reflected both in referral decisions and in interactions with patients during psychosocial assessments, where practitioners redirected patients toward self-management, coping strategies, or informal support when further service input was unavailable. Practitioners reported moral distress and frustration associated with denying access to care and delivering “bad news” under conditions of high demand and limited resources. | Bergen, Lomas et al.; Quinlivan et al. | High confidence | No or very minor concerns on methodological limitations, no or very minor concerns on coherence, no or very minor concerns on adequacy, minor concerns on relevance (UK context only). |
| 10 | **Absence of timely support in the community attributed to repeat attendance to ED but support needed for patients beyond the remit of ED MHPs.**  MHPs described a cycle of repeated attendances by patients presenting with self-harm or suicidal ideation which they attributed to the absence of timely and effective community-based treatment. MHPs reported recognising trauma and psychosocial factors as underlying these presentations but described addressing such issues as beyond the remit of emergency care and unfeasible given limited resources. As a result, mental health support in ED setting was characterised as providing short-term or “band-aid” responses, with MHPs anticipating that patients would return due to unresolved underlying difficulties. | McCarthy et al.; O'Keeffe et al.; Quinlivan et al.; Rheinberger et al. | High confidence | No or very minor concerns on methodological limitations, no or very minor concerns on coherence, no or very minor concerns on adequacy, minor concerns on relevance (only high-income Western context represented) |
| 11 | **Experiencing feelings of futility and powerlessness linked to desensitization and burnout but no formal mental health support for MHPs.**  MHPs described repeat ED attendances and the inability to offer adequate care as taking a significant emotional toll, contributing to feelings of futility, powerlessness, and growing cynicism. MHPs reported desensitisation and emotional detachment as protective responses to burnout. At the same time, they described limited availability of formal pastoral or mental health support within services, linking this to poor staff retention, and reported relying on informal peer support and teamwork to cope with the emotional demands of their work. | McCarthy et al.; O'Keeffe et al.; Quinlivan et al.; Rheinberger et al. | High confidence | No or very minor concerns on methodological limitations, no or very minor concerns on coherence, no or very minor concerns on adequacy (3 studies contributed to review finding with relatively thick data. 1 study contributed thin data), minor concerns on relevance (only high-income Western context represented) |
| **Theme 4: Strategies to facilitate access and extending care to overcome challenges in ED** | | | | |
| 12 | **Multidisciplinary team input and clinical documentation facilitate decision-making and referrals.**  MHPs described how access to multidisciplinary input, including consultants and clinical psychologists, supported decision-making and facilitated referrals to secondary mental health services. Availability was contingent on local service configuration and commissioning arrangements. Clinical documentation was also informed assessments and MHPs expressed a need for more detailed and meaningful notes to support onward-care decisions. | Chunduri et al; Quinlivan et al. | High confidence | No or very minor concerns on methodological limitations, no or very minor concerns on coherence, minor concerns on adequacy (only 2 studies contributed relatively thick data), minor concerns on relevance (only high-income Western context represented) |
| 13 | **Tailoring documentation to align with service eligibility can facilitate access.**  When negotiating referrals to secondary or specialist mental health services following emergency assessments, MHPs described actively advocating for patients by tailoring referral documentation and clinical language to align with service eligibility thresholds. MHPs reported using targeted phrasing to increase the likelihood of acceptance and described adjusting how patient risk was presented across stages of the care pathway, including exaggerating risk to pursue admission but this could lead to situations of later downplaying risk to justify discharge if referrals were rejected. | Chunduri et al; Quinlivan et al. | Moderate confidence | No or very minor concerns on methodological limitations, no or very minor concerns on coherence, moderate concerns on adequacy (1 study contributed to review finding with relatively thick data. 1 study contributed thin data), minor concerns on relevance (only high-income Western context represented). |
| 14 | **Novel outpatient clinic models can extend care beyond ED.**  MHPs described a novel outpatient clinic model that extended the care pathway beyond EDs, allowing more comprehensive psychosocial assessments than were possible during acute presentations. MHPs reported that this model enabled them to strengthen care plans, maintain follow-up or crisis re-contact after ED discharge, and feel more connected to patients’ longer-term recovery, which they perceived as helping to reduce repeated emergency attendances. However, they also described the model as uncommon and operating with narrowly defined eligibility criteria, meaning that some patients continued to experience gaps in care following emergency department discharge. | Quinlivan et al. | Moderate confidence | No or very minor concerns on methodological limitations, coherence not applicable as only one study, minor concerns on relevance (only UK context represented), moderate concerns on adequacy (1 study contributed to review finding with relatively thick data), |
| **Theme 5: Potential for training to counter negative attitudes and stereotypes that are still prevalent** | | | | |
| 15 | **Stigmatising beliefs persist despite describing their approaches as empathetic and trauma-informed.**  MHPs described their approaches as empathetic and trauma-informed, recognising self-harm as a coping mechanism and expressing concern that abrupt discouragement could be harmful when it was a patient’s primary way of managing distress. MHPs also reported that explaining the role of trauma in self-harm could help patients feel understood and experience relief. At the same time, negative language and stereotypes about self-harm persisted, including framing behaviours as attention-seeking, a “cry for help,” or a matter of personal choice. These stigmatising framings were described as embedded within workplace cultures and reinforced through senior staff attitudes. | McCarthy et al; Murphy et al. | Moderate confidence | No or very minor concerns on methodological limitations, no or very minor concerns on coherence, minor concerns on adequacy (2 studies contributed to review finding with relatively thick data. 1 study contributed thin data), moderate concerns on relevance (1 study had various settings (in-patient units, community settings and Emergency Department) and only high-income Western context represented). |
| 16 | **Expressed need for greater training to increase confidence and potential to challenge stigmatising attitudes.**  MHPs described a strong need for training to improve care for people presenting with self-harm or suicidal distress in emergency settings. MHPs reported wanting to update their skills and knowledge, linked training to increased confidence in their role, and described education as enabling them to challenge stigmatising attitudes in themselves and colleagues. They also described limited feedback on patient outcomes as a missed learning opportunity for informing future care. At the same time, MHPs reported that existing professional training, including nursing education, was often too general and insufficiently tailored to the realities of emergency mental health work, leaving them feeling unprepared for practice in these settings. | McCarthy et al; Murphy et al. | Moderate confidence | No or very minor concerns on methodological limitations, no or very minor concerns on coherence, minor concerns on adequacy (2 studies contributed to review finding with relatively thick data. 1 study contributed thin data), moderate concerns on relevance (1 study had various settings (in-patient units, community settings and Emergency Department) and only high-income Western context represented). |

*For further details see Supplementary Materials 4
